# Notes of the Week

**Published:** 1921-04-09

**Authors:** 


### NOTES OF THE WEEK.

### HOSPITALS AND APPROVED SOCIETIES FUNDS.

At the conference on national health held last Saturday under the auspices of the Faculty of Insurance, Ltd., at the Central Hall, Westminster, the vexed question as to the nature of approved societies and additional benefits was debated. Sir Kingsley Wood, of course, took the same view as the Minister of Health with regard to the claim of the voluntary hospitals, and he was supported by Sir Thomas Neill, of the Consultative Council of the Health Insurance Approved Societies, who said that it was clear that, inasmuch as the hospitals cured the members of the societies of their ailments, they therefore saved considerable sums which otherwise would have to be paid in sickness benefit. Mrs. Handel Booth urged that the hospital system was not sufficiently complete throughout the country, and that villagers had difficulty in getting into hospitals. She was averse to handing over a lump sum, but suggested as an alternative that if members of approved societies received treatment a suitable donation to the hospital should be made in each case. The ideal method would be, of course, a sufficient payment for each member, and Mrs. Booth's suggestion, taking it that the donation would vary according to the length of treatment, is quite a good one.

### THE FEDERATION'S PROGRESS.

At a meeting of the Executive Council of the Federation of Medical and Allied Societies held on April 5 it was announced that its incorporation had been satisfactorily completed. Sir Berkeley Moynihan, K.C.M.G., C.B., M.S., of Leeds, was elected the first President, and Sir Malcolm Morris, K.C.V.O., F.R.C.S.E., the Chairman of Council, was elected Vice-President. In view of the increasing activities of the Federation, and in accordance with its Articles of Association, the Executive Council now delegates the control of its various sections to the following branch councils: (1) A Medical Council, (2) a Council of Allied Professions, and (3) a Citizens' Council. Each society is now to have a representative on the Council appropriate to its professional interests. These Councils will meet quarterly as the Executive Council, and at other times in joint conference. It is anticipated that the offices will be transferred to more suitable premises at a very early date, where it will be possible to provide the constituent societies with a conference-hall and certain secretaries and other advantages. Current literature dealing with the public aspect of medicine is also always to be available. The meeting had many matters under its consideration, including reports as to recent action undertaken on behalf of the societies in regard to the Dangerous Drugs Act Regulations and the change of Minister at the Health Ministry. The British Association of Radiology and Physiotherapy was elected to membership. We heartily congratulate the Federation upon the progressive spirit in which the work of development is being undertaken.

### MILK FOR MOTHERS.

A recent Order of the Minister of Health greatly restricts the powers of local authorities with regard to the supply of milk to expectant and nursing mothers and children which they possessed under the Milk Orders of 1919. In future authorities are obliged to obtain the sanction of the Minister to any schemes they are proposing. The step has been found necessary because expenditure has largely exceeded what was intended. Schemes must be framed in a way that supply of milk at less than cost must be restricted to necessitous cases of nursing mothers or expectant mothers in the last three months of pregnancy, and children up to three years of age, except in exceptional cases, where the age may be up to five years. The quantity must not exceed ordinarily one pint a day per person, but in certain instances a pint and a-half may be given. Necessity must be certified by the medical officer of health or the medical officer of a Centre. Under the new arrangement it is probable that the ratepayer will benefit materially, and genuinely suitable cases will suffer not at all.

### THE UNEMPLOYED MINER.

The coal strike gives special interest to the affairs of the miners' hospital at Redruth. Because of a deficiency last year of £700 the hospital committee recommended a reduction in the number of beds, and called a general meeting to consider also a plan for charging ten shillings a week toward the cost of each patient's maintenance. Dr. Mayne then put forward a special plea for the Cornish miner who has for some year contributed weekly from his wages. If, said Dr. Mayne, such a man is injured, he would lose his unemployment pay, and under the insurance scheme would receive fifteen shillings a week. If ten shillings were deducted toward the cost of his maintenance in hospital, his wife and family would have only five shillings a week on which to live. There must surely be a misapprehension here: an injured miner is not entitled to 15s. or any other sum from the National Health Insurance funds, but is entitled to a much higher rate of compensation from his employers. It was eventually decided that married miners, with dependants, who have been contributors to the hospitals, should be given special consideration at the discretion of the committee.

### A SECRETARY ON PAID ORGANISERS.

The reopening of the North Staffordshire Heath Memorial Convalescent Home for Men, Llanfairfechan, coincides unfortunately with the retirement of Mr. Sandland from the secretaryship after many years' service. Mr. Philip Elliott, chairman of the committee, said that Mr. Sandland has been out of pocket for the assistance he has required, and that on many grounds they ought to be sincerely thankful to him. In reply Mr. Sandland urged the need for a paid organising secretary. If he had not had a staff of clerks, he could not have done the work, or carried through the scheme to ensure regular contributions from the factories. The appointment of a successor will lead to the settlement of this matter, and Mr. Sandland's experience is a characteristic example of the unselfishness with which voluntary work is done.

### TWO LARGE CHARITABLE DISTRIBUTIONS.

The residue of the estate of the late Mr. Albert Ernest Watson, a director of the Maypole Dairy Company, has been left to King Edward's Hospital Fund, the British Red Cross Society, Dr. Barnardo's Homes, and the Salvation Army. It is expected that about £250,000 will be distributed. Through its British branch the National Allied Relief Committee of the U.S.A. has distributed nearly £3,000 contributed by American citizens of all ages, classes, and creeds for charitable purposes in the British Isles as a token of admiration for Great Britain's part in the late war. King's College Hospital is one of the participants.

### THE ST. MARY'S UNITS.

We are more than glad to hear that the University Grants Committee has recommended a grant towards clinical units in Medicine and Surgery at St. Mary's Hospital Medical School. These grants will be parallel to those made to the four already recognised schools, and we congratulate the St. Mary's authorities upon the possibility before them. Earlier it had been decided to discontinue the units at this hospital until State contribution to their maintenance could be made. A petition was then signed by practically every student in the school asking that the units should be carried on. We already know the actual value of this teaching method, and the St. Mary's students have now given a remarkable proof of its popularity.

### CONSULTANTS FOR ST. PANCRAS POOR-LAW HOSPITAL.

The lead given by Paddington Infirmary in co-operation with St. Mary's Hospital is, we are informed, being followed at the St. Pancras Infirmary, where students of the London School of Medicine for Women will attend for clinical experience. The Medical Superintendent, Dr. C. Thackeray, M.D., has given considerable thought to the necessary arrangements, and the Guardians have now agreed to appoint consultants to attend at the infirmary to give advice on certain cases. Mr. Norbury, F.R.C.S., has been appointed as consulting surgeon, and Dr. E. Bolton as consulting gynæcologist. We hope similar co-ordinating schemes are in hand at other Poor-law infirmaries, and we shall be glad to hear of their development.

### ST. THOMAS'S HOSPITAL.

On inquiry at St. Thomas's Hospital it was ascertained from Mr. G. Q. Roberts, C.B.E., the Secretary, that the result of the £100,000 appeal is, up to the present, most disappointing, the amount received being considerably short of £5,000. The appeal would have been issued earlier, but was postponed so as not to clash with that of St. Bartholomew's, but Mr. Roberts recalled the fact, which doubtless most people have forgotten, that St. Thomas's is itself, by origin, a City Hospital, having been until 1866 located near London Bridge. St. Thomas's was a Military Hospital during the war, and its present indebtedness is claimed to be in part attributable to the inadequate allowances made by the Government. Extreme needs such as those of St. Thomas's require extreme methods of appeal, but up to now the cry of this great hospital has not been sufficiently insistent to reach the ears of the multitude, who are much pre-occupied by other matters and are unlikely to respond unless an appeal is brought very prominently before them.

### "THE MUNDY HOSPITAL."

The Mayor of Derby, a medical man, Dr. R. Laurie, was doubly fitted to preside at the recent gathering of subscribers to the Derbyshire Hospital for Women, when plans for the desired extension were discussed. Because of the help given by Mrs. Mundy, the generous donor of the premises to be adapted, Dr. Laurie asked if the hospital could not be appropriately called "The Mundy Hospital." It could, if it had not been called first the Derbyshire Hospital for Women. Founders' names make good names for hospitals, but once a territorial title has been chosen to revert to the surname of a benefactor is unusual.

### DENTISTS FOR THE NAVY.

New regulations have just been issued for admission to the Royal Naval Dental Service. They are more than welcome, as dentistry has been not a little neglected among sailors. After experiences in the war the reform became an obvious necessity. Candidates must be fully-qualified dentists and registered under the Dentists Act. They must, after passing the preliminary tests as to fitness, etc., present themselves for examination, and, if successful, will receive appointment in accordance with order of merit. Surgeon-Lieutenants will receive as annual full pay £401 to £492, Surgeon-Lieut.-Commanders £593 to £730, and Surgeon-Commanders £775 to £912. Speaking generally, the officers will also be eligible for allowances on the same scale as for medical officers of the same rank.

### WHAT CONTRIBUTION SCHEMES CAN DO.

Norwich, which is one of the places where hospital finances has been a severe difficulty, has made remarkable progress with its Saturday and Sunday Fund. The sum for division in the report for 1920 was £6,417, or more than twice the total of the preceding year. Every item showed an increase, and there seems no doubt that the total for 1921 will be much bigger because of the increasing number of firms which have adopted the contribution scheme. While not exaggerating this progress, it can at all events be said that, if expenses have trebled, the income of hospitals where such schemes have been started are on the way to doubling themselves. The Mansfield Hospital, Notts, is another institution for which the income from contributions has doubled during the past year alone.

